# Oral health status and associated lifestyle behaviors in a sample of Iranian adults: an exploratory household survey

**DOI:** 10.1186/s12903-020-01072-z

**Published:** 2020-03-19

**Authors:** Leila Jahangiry, Raziollah Bagheri, Fatemeh Darabi, Parvin Sarbakhsh, Mohammad Mehdi Naghibi Sistani, Koen Ponnet

**Affiliations:** 1 Health Education and Health Promotion Department, School of Public Health, Tabriz University of Medical Sciences, Tabriz, Iran; 2 Tabriz Health Services Management Research Center, Tabriz University of Medical Sciences, Tabriz, Iran; 3 Health Education & Promotion Department, Department of Public Health, Asadabad School of Medical Sciences, Asadabad, Iran; 4 Epidemiology and Biostatistics Department, School of Public Health, Tabriz University of Medical Sciences, Tabriz, Iran; 5 Oral health research center, Health Research Institute, Babol University of Medical Sciences, Babol, Iran; 6 Faculty of Social Sciences, imec-mict-Ghent University, Ghent, Belgium

**Keywords:** Oral health status, Household survey, Adults, Older population

## Abstract

**Background:**

Poor oral hygiene can lead to serious diseases, such as periodontitis, tooth decay, pain and discomfort in teeth or gums, infections, and loss of teeth. In Iran, adults aged 50 y and older are a high-risk group for oral health problems, and this age group will grow in the coming decades. Despite increasing attention on healthy aging, there is relatively less emphasis on oral hygiene and health-related problems. The present study investigated the oral health status of Iranian adults using the oral health self-assessment questionnaire (OHQ) developed by the World Health Organization (WHO).

**Methods:**

A population-based household survey of a sample of adults aged 18–65 y was conducted. In this study, the participants were recruited between May and October 2016 in Tabriz, Iran, and the study population was sampled using a multi-stage cluster sampling design. The WHO’s OHQ for adults was used for measuring oral health status and oral hygiene behavior.

**Results:**

In total, 2310 respondents completed the survey. The mean age (SD) of the participants was 41.6(23.4) y. Males accounted for 48.8% of the participants. Of the 2310 respondents,187 (8.1%) individuals were edentulous, 152(20.7%) of whom were aged 51–65 y. Furthermore, 72.3% of those aged 51–65 y were dentate, and 50% of adults aged 51–65 y said they had 20 or more teeth. About one-third of the participants reported that they did not brush their teeth daily (23% of those aged 18–35 y,35.9% of those aged 36–50 y, and 44.6% for those aged 51–65 y). In the sample, 39.4% of individuals aged 18–35 y,34.1% of individuals aged 36–50 y, and 26.6% of individuals aged 51–65 y had visited a dentist less than 6 month ago. One-third of the participants consumed sweets and sugary drinks daily.

**Conclusions:**

Although the majority of Iranian adults considered their oral health status good, only a small percentage of the sample visited their dentist regularly. Furthermore, visits to the dentist declined in accordance with increasing age, a time when the incidence of oral health problems may increase. Poor oral health may increase the risk of adverse health outcomes, particularly among the aging population.

## Introduction

Poor oral hygiene has a significant impact on oral health and can give rise to various problems, such as periodontitis, tooth decay, tooth or gum pain and discomfort, and infection and loss of teeth. Current evidences show that poor oral hygiene is cause of biofilm accumulation and that relatively specific group of indigenous oral bacteria is cause of plaque-induced inflammatory periodontal diseases [1]. Poor oral health can result in additional complications, included swallowing, chewing, and speech difficulties, and these, in turn, can affect sleep quality and work productivity [2, 3].

There is strong evidence that untreated oral diseases and disorders not only negatively affect general health but also increase the probability of diabetes and cardiovascular diseases [4, 5]. A meta-analysis of follow-up studies estimated that poor oral health and periodontal diseases may increase the risk of such diseases by approximately 20% [6]. Other studies indicated that periodontal diseases significantly increased the risk of myocardial infarctions [6, 7].

Natural tooth loss, dental plaque accumulation, and inflammation of gingival tissues increase with age [8]. The aging population in Iran is set to increase dramatically in the coming decades [9]. According to the World Health Organization (WHO), nearly 10% of the Iranian population is older than 60 y, and this will rise to 21.7% by 2050 [10–12]. As a result of this aging population, the prevalence of chronic diseases, especially health and oral health problems, will increase, which has implications for health care service use and costs [13, 14]. Although increasing attention is paid to healthy aging, there is relatively little emphasis on oral health. Some years ago, the WHO recommended conducting household surveys of adults to gather epidemiological information on the oral health status and dental caries patterns of different age groups, with the findings utilized to draw up appropriate and tailored oral health care [15].

The aim of this study was to investigate the oral health status (the number of natural teeth, pain or discomfort on teeth, having dentures) and oral health behaviors (e.g., using toothbrush, dental flossing, and toothpaste containing fluoride) of people living in urban areas in Tabriz, Iran, and to determine demographic (age and gender) differnces between them through a houshold survey. Such information is essential for future prevention programs and oral health interventions. In the present study, we conducted a household survey to investigate the oral health status of adults in Tabriz, Iran. Specifically, we administered the WHO’s oral health self-assessment questionnaire (OHQ) to a sample of Iranian adults of different ages.

## Methods

### Study design and population

This cross-sectional study focused on adults aged 18–65 y who were recruited between May and October 2016 in Tabriz. A population-based household survey was conducted to recruit a sample of adults from the community. The eligibility criteria were as follows: a) living in the study area and being at home at the time of data collection and (b) not having a diagnosis of mental or cognitive disorders. Written informed consent was obtained from each participant.

### Sampling and sample size

The population of Tabriz was estimated to be 980,000 (aged 18–65 y) in 2016 [16]. To estimate the oral health status of 50% (edentulous) of the population, according to the study [9] a likely sample proportion was considered 50%, then, with a 95% confidence, an error of 1.8% and finite population number (980,000), the sample size was estimated (*n* = 2956). We rounded it to 3000 in practice. For sampling, the study population was sampled using a multi-stage cluster sampling design [17]. In the first sampling stage, the city was divided into four areas (West North, West South, East North, and East south) based on socio-economic status. In the next stage, one health care centers (HCC) was randomly selected from each area (in total four HCCs from 16 HCCs). Because all people’s health records were available in the HCCs, they are used in the sampling as first sampling units. In the next stage, 25% of street blocks in the area of the selected four HCCs were randomly selected. Determined sample size (3000 persons) was proportionally divided into four area. For each block, every 11th household in the block was then contacted, and the suitability of the household members for inclusion in the study was assessed. Finally, in each household, eligible individuals were asked to participate in the study. In total, 690 people refused to participate in the study. Sampling in the blocks was continued until the required sample size specified for that block was reached. Four trained researchers conducted the interviews. Interviewers were public health students working as public health researchers and having at least 2 years of experience as an interviewer in survey-based health researches. They received three 90- min training sessions on successful performance of completing the study questionnaire. Their first ten interviews were qualitatively evaluated by 4 experienced researchers, to guarantee the quality of the interviews. In total, 2310 respondents completed the survey (a 77% response rate).

### Data collection and questionnaire

The WHO’s OHQ for adults was used for measuring the oral health status and oral health behaviors of the participants [17]. The WHO’s OHQ consists of 16 variables. Four questions are used to assess oral health status: (a) self-reported number of teeth present (0 = no natural teeth, 1 = 1–9 natural teeth, 2 = 10–19 natural teeth, and 3 = 20 natural teeth or more),; (b) experience of oral/dental pain/discomfort (yes/no); (c) wearing of removable dentures (partial/full upper/full lower; yes/no); and (d) self-assessment of the status of teeth and gums (ranging from 1 = excellent to 6 = very poor). Three items are used to assess oral health related behaviors: (a) frequency of perform oral hygiene (ranging from 1 = never to 6 = once a day); (b) use of aids/tools for oral hygiene (e.g., toothbrush, wooden toothpick, dental floss; yes/no); and (c) use of a fluoride- or nonfluoride toothpaste (yes/no). Two questions are related to the frequency of dental visits, with scores ranging from 1 (less than 6 mo ago) to 5 (5 y ago or longer), and the reasons for the dental visit(s) (e.g. consultation/advice, treatment/follow-up treatment, and dental or oral pain/discomfort). One question assesses reduced quality of life (QoL) due to 12 oral problems (e.g., difficulty biting or chewing food), with the responses scored as 0 = don’t know, 1 = no, 2 = sometimes, 3 = fairly often, and 4 = very often. One question examines the consumption of sugary foods (e.g., biscuits and cream cakes) and drinks (e.g., tea with sugar) and fresh fruit consumption, with the answers scored as follows: 1 = never/seldom, 2 = several times a month, 3 = once a week, 4 = several times a week, 5 = every day, and 6 = several times a day. One item assesses tobacco use (cigarettes, pipe, cigar, snuff, and tobacco chewing) and frequency (1 = never/seldom, 2 = several times a month, 3 = once a week, 4 = several times a week, 5 = every day, and 6 = several times a day). The last question assesses the consumption of alcohol (usually the number of drinks per day) during the past 30 d (ranging from 0 = less than 1 drink to 5 = 5 or more drinks). The remaining questions relate to the socio-demographic characteristics of the sample in terms of age (18–35, 36–50, and 51–65 y) and sex (male or female).

The validity and reliability of the WHO’s OHQ was assessed based on standardized cross-cultural translation guidelines [18]. The English version of the WHO’s OHQ was translated into Persian. Two native Persian translators unrelated to the study translated the OHQ. Any disputes were resolved by consensus.. Two professional translators then back translated the OHQ from Persian to English. The translators and researchers checked and agreed on the final Persian version. Content validity was determined in a pilot study of 20 individuals [19]. Following feedback on the scale, changes were made to the wording of some items. The Persian version of the WHO’s OHQ was then evaluated by an expert panel (three health educationists and two dentists).

### Statistical analysis

Statistical analyses were performed using the Statistical Package for Social Science, Version 18 for Windows (SPSS Inc., Chicago, IL, USA). Normality of the data was analyzed using the Kolmogorov–Smirnov test. Discrete variables are presented with numbers and percentages. According to the sampling design, weight of each observation was determined and this weight was used in data analysis. Chi square test was used to assess relationship between categorical variables. In all tests, a value of *p* < 0.05 was considered statistically significant.

## Results

### Demographic characteristics and oral health status

The mean age (SD) of the 2310 participants was 41.6 (23.4) y. Males accounted for 48.8% of the study population. In the sample, 187 (8.1%) participants were edentulous, and 152 (20.7%) of these participants were aged 51–65 y. In addition, 72.3% of the participants were dentate (i.e. 20 or more teeth). The rate was 92.7% for adults aged 18–35 y, 71.3% for adults aged 36–50 y, and 49.4% for adults aged 51–65 y.

In the study, 61.2% of all participants reported experiencing dental and oral pain or discomfort in the previous 12 mo. Table 1 presents an overview of the oral health status of the participants in the different age groups (18–35 y, 36–50 y, and 51–65 y). The results revealed significant between-group differences, with those aged 18–35 y (71.2%) reporting significantly more dental/oral pain or discomfort as compared with that of the individuals aged 36–50 y (55.8%) and 51–65 y (54.8%). In total, 359 participants (15.5%) had a removable partial denture, of whom 9% were aged 18–35 y, 16.6% were aged 36–50 y, and 21.8% were aged 51–65 y.

**Table 1:** Dental health status of the participants

Variables	Age (y); *n* (%)			*p*-value	Total		*p*-value
Number of natural teeth	**18–35**	**36–50**	**51–65**	< 0.0001	**Male**	**female**	0.553
No natural teeth	0 (0)	35 (4.9)	152 (20.7)		93 (49.7)	94 (50.3)	
1–9	0 (0)	21 (2.9)	155 (21.1)		87 (49.4)	89 (50.6)	
10–19	63 (7.3)	150 (20.9)	64 (8.7)		124 (44.8)	153 (55.2)	
20 teeth or more	795 (92.7)	513 (71.3)	362 (49.4)		824 (49.3)	846 (50.7)	
Dental/oral pain or discomfort during the last 12 mo (yes) (yes)	611 (71.2)	401 (55.8)	402 (54.8)	< 0.0001	657 (46.5)	757 (53.5)	0.004
Presence of removable dentures							
Partial removable denture	80 (9.3)	119 (16.6)	160 (21.8)	< 0.0001	174 (48.5)	185 (51.5)	0.881
Full upper denture	9 (1)	57 (7.9)	187 (25.5)	< 0.0001	127 (50.2)	126 (49.8)	0.645
Full lower denture	4 (0.5)	44 (6.1)	157 (21.4)	< 0.0001	103 (50.2)	102 (49.8)	0.672
Status of dental health				0.061			0.141
Good	526(60.8)	475 (66.2)	424 (57.8)		722 (50.7)	703 (49.3)	
Average	265 (30.5)	138 (19.1)	188 (31.8)		275 (46.5)	53.5 (316)	
Poor	49 (5.7)	94 (13.1)	88 (38.1)		106 (45.9)	125 (54.1)	
Status of gum health				0.050			
Good	310 (39.2)	292 (46.6)	258 (40.0)		417 (48.5)	443 (51.5)	0.202
Average	126 (16.0)	79 (12.5)	96 (14.2)		137 (45.5)	164 (54.5)	
Poor	354 (44.7)	256 (40.9)	289 (44.8)		460 (51.2)	439 (48.8)	

With regard to self-reported dental health status, 63.5% (*n* = 1425) reported good dental health. There was no significant difference among the three age groups in terms of self-reported health status.

The tooth brushing and cleaning habits of the three age groups are shown in Table 2. About one-third of the participants reported that they did not brush their teeth daily (23% in the 18- to 35 y -old age group, 35.9% in the 36- to 50 y-old age group, and 44.6% in the 51- to 65 y-old age group). In terms of tooth cleaning habits, 62% reported using a toothbrush, 24% reported using dental floss, and 62% reported using toothpaste.

**Table 2:** Frequency and percentage of dental hygiene habits (tooth cleaning) of the study sample

Variables	Age (y), ***n*** (%)			***p***-value	Total		***p***-value
	18–35	36–50	51–65		Male	female	
**Tooth cleaning**				0.055			0.104
Less than once a day	202 (23.6)	259 (35.9)	328 (44.6)		404 (51.2)	385 (48.8)	
Once a day or more	656 (76.4)	460 (64.1)	405 (55.4)		724 (47.6)	797 (52.4)	
**Toothbrush**	527 (61.4)	460 (62.7)	405 (62.3)	0.835	673 (46.9)	763 (53.1)	0.015
**Wooden toothpicks**	146 (17)	155 (21.6)	158 (21.6)	0.03	222 (48.4)	237 (51.6)	0.824
**Plastic toothpicks**	6 (0.7)	7 (1)	21 (2.9)	0.01	15 (44.1)	19 (55.9)	0.580
**Dental floss**	213 (24.8)	175 (24.3)	164 (22.4)	0.01	243 (44.0)	309 (56.0)	0.010
**Nonfluoride toothpaste**	527 (61.4)	460 (62.7)	405 (62.3)	0.570	1020 (48.8)	1071 (51.2)	0.880
**Fluoride-containing toothpaste**	315 (36.4)	241 (33.4)	245 (32.7)	0.068	823 (48.8)	864 (51.2)	0.068

Table 3 provides information on dental care visits among the three age groups and the reasons for these visits. In the study, 39.4, 34.1, and 26.6% of adults aged 18–35 y, 36–50 y, and 51–65 y, respectively, had visited a dentist less than 6 mo. The most frequent reason (50.9%) for visiting the dentist was dental or oral pain or discomfort.

**Table 3:** Frequency of dental visits and the reasons for these visits

Variable				*p*-value	Total		*p*-value
	Age (y), *n* (%)				Male	Female	
	18–35	36–50	51–65				
Dental visit				< 0.001			0.517
Less than 6 mo ago	274 (32.0)	237 (33.3)	185 (26.0)		347 (49.9)	349 (50.1)	
More than 6 mo ago	584 (68.0)	482 (66.7)	548 (74.0)		781 (48.4)	833 (51.6)	
Reason of last visit to the dentist				< 0.001			0.002
Consultation/advice	36 (4.2)	41 (5.7)	28 (3.8)		68 (64.8)	37 (35.2)	
Dental/oral pain (teeth, gums/mouth)	433 (50.5)	291 (40.5)	451 (61.5)		581 (49.4)	594 (50.6)	
Treatment	291 (33.9)	247 (34.4)	186 (25.4)		351 (48.5)	373 (51.5)	
Routine check-up	92 (10.7)	133 (18.5)	63 (8.6)		119 (41.3)	169 (58.7)	

Table 4 provides an overview of the oral health problems of the study population during the last year. The most frequent oral health issues reported by adults aged 18–35 y were cosmetic concerns related to the appearance of teeth. In contrast, the most frequent oral health problem among adults aged 36–50 y and 51–65 y was mastication difficulty (43.5 and 54%, respectively).

**Table 4:** Frequency and percentage of Oral health problems experienced during the previous 12 mo

Variable	Age (y), *n* (%)				Total		
	18–35	36–50	51–65	*p*-value	Male	Female	*p*-value
Dental problems during the past 12 mo							
Biting difficulty	238 (28.5)	262 (38.2)	320 (47.3)	< 0.0001	407 (49.6)	413 (50.4)	0.561
Difficulty chewing food	208 (24.8)	297 (43.5)	387 (54.0)	< 0.0001	425 (47.6)	467 (52.4)	0.458
Speech/word pronouncing difficulties	52 (6.5)	106 (15.4)	65 (8.9)	< 0.0001	111 (49.8)	112 (50.2)	0.725
Dry mouth	155 (19.6)	85 (12.6)	54 (8.1)	< 0.0001	144 (49.0)	150 (51.0)	0.975
Felt embarrassed due to appearance of teeth	257 (30.6)	187 (27.6)	155 (22.1)	< 0.0001	298 (49.7)	301 (50.3)	0.737
Felt tense due to teeth or mouth	354 (44.4)	280 (40.9)	243 (34.8)	< 0.0001	459 (52.3)	418 (47.7)	0.014
Smiling avoidance because of concerns about teeth or mouth	205 (25.5)	172 (25.4)	123 (18.1)	< 0.0001	224 (44.8)	276 (55.2)	0.056
Sleep interruption	129 (16.4)	90 (13.2)	115 (17.6)	0.001	167 (50.0)	167 (50.0)	0.580
Days off work	165 (19.7)	79 (11.7)	63 (9.3)	0.001	161 (52.4)	146 (47.6)	0.167
Difficulty doing usual activities	119 (14.8)	104 (15.0)	127 (18.3)	0.001	159 (45.4)	191 (54.6)	0.208
Felt less tolerant of spouse or close people	52 (6.6)	61 (8.1)	69 (10.4)	0.048	78 (42.9)	104 (57.1)	0.089
Reduced tolerance of spouse or close friends	104 (13.0)	118 (17.3)	100 (14.8)	0.062	155 (48.1)	167 (51.9)	0.884

Information on lifestyle behaviors, including dietary habits (e.g., the consumption of fresh fruit, sweets [biscuits and cakes], and sugary drinks) and tobacco and alcohol consumption, is provided in Table 5. Only 5.7% of the participants consumed fresh fruits several times a day, and 85% of the participants consumed fresh fruits several times a week. About one-third (35%) of the participants consumed biscuits, 57.1% consumed cakes, 38% consumed jam or honey every day, and half of them consumed tea with sugar each day. In the sample, 88% of the participants reported never or seldom smoking, and 6% reported consuming alcohol several times a week.

**Table 5:** Frequency and percentage of lifestyle behaviors related to oral health

Eating or drinking the following foods	Age; *n* (%)			*p*-value			
	18–35	36–50	51–65		Male	Female	*p*-value
Fresh fruits				0.093			0.037
Often	757 (88.2)	613 (85.3)	619 (84.4)		953 (84.5)	1036 (87.6)	
Occasionally	86 (10.0)	90 (12.5)	89 (12.1)		149 (13.2)	116 (9.8)	
Seldom	15 (1.7)	16 (2.2)	25 (3.4)		26 (2.3)	30 (2.5)	
Biscuits				< 0.001			0.720
Often	399 (46.5)	265 (36.9)	170 (23.2)		415 (36.8)	419 (35.4)	
Occasionally	322 (37.5)	324 (45.1)	412 (56.2)		515 (45.7)	543 (45.9)	
Seldom	137 (32.8)	130 (31.1)	151 (20.6)		198 (17.6)	220 (18.6)	
Sweet cakes, pies, buns				< 0.001			0.646
Often	565 (65.9)	406 (56.5)	352 (48.0)		657 (58.2)	666 (56.3)	
Occasionally	203 (23.7)	198 (27.5)	282 (38.5)		327 (29.0)	356 (30.1)	
Seldom	90 (10.5)	115 (16.0)	99 (13.5)		144 (12.8)	160 (13.5)	
Jam or honey				< 0.001			0.004
Often	413 (48.1)	281 (39.1)	209 (28.5)		480 (42.6)	423 (35.8)	
Occasionally	325 (37.9)	331 (46.0)	366 (49.9)		473 (41.9)	549 (46.4)	
Seldom	120 (14.0)	107 (14.9)	158 (21.6)		175 (15.5)	210 (17.8)	
Chewing gum containing sugar/sweets /candy				< 0.001			0.924
Often	109 (12.7)	73 (10.2)	69 (9.4)		125 (11.0)	126 (10.7)	
Occasionally	308 (35.9)	195 (27.1)	227 (31.0)		353 (31.3)	377 (31.9)	
Seldom	441 (51.4)	451 (62.7)	437 (59.6)		650 (57.6)	679 (57.4)	
Lemonade, Coca Cola or other soft drinks				< 0.001			0.40
Often	231 (26.9)	719 (17.9)	439 (19.0)	< 0.001	231 (20.5)	208 (17.6)	
Occasionally	365 (30.5)	258 (35.9)	305 (41.6)		521 (46.2)	525 (44.4)	
Seldom	262 (30.5)	258 (35.9)	305 (41.6)		376 (33.3)	449 (38.0)	
Tea with sugar							0.045
Often	538 (62.7)	409 (56.9)	335 (45.7)	< 0.001	644 (57.1)	638 (43.)	
Occasionally	195 (22.7)	167 (23.2)	229 (31.2)		294 (26.1)	297 (25.1)	
Seldom	125 (14.6)	143 (19.9)	169 (23.1)		190 (16.8)	247 (20.9)	
Coffee with sugar				< 0.001			0.930
Often	288 (33.6)	102 (14.2)	111 (15.1)		241 (21.4)	260 (22.0)	
Occasionally	253 (29.5)	265 (36.9)	24 (34.0)		375 (33.2)	392 (33.2)	
Seldom	317 (36.9)	352 (49.0)	37 (50.9)		512 (45.4)	530 (44.8)	
Tobacco use				< 0.001			< 0.001
Often	55 (6.4)	82 (11.4)	68 (9.3)		131 (11.6)	74 (6.3)	
Occasionally	28 (3.3)	17 (2.4)	7 (1.0)		28 (2.5)	24 (2.0)	
Seldom	775 (90.3)	620 (86.5)	658 (89.8)		969 (85.9)	1084 (91.7)	
Alcohol use				0.409			0.013
Often	0.0	0.0	0.0		0	0	
Occasionally	23 (2.8)	20 (3.0)	27 (4.0)		44 (4.2)	26 (2.3)	
Seldom	794 (97.2)	653 (97.0)	653 (96.0)		1004 (95.8)	1096 (97.7)	

Note: Often (Several times a day, Every day, and Several times a week); Occasionally (Once a week and Several times a month); Seldom (Seldom and never)

## Discussion

This household survey aimed to determine the oral health status of adults living in Tabriz, Iran. This oral health survey revealed that about 72% of the study population had 20 or more teeth but that only 50% of those aged 51–65 y had 20 teeth or more. A similar study conducted in Iran among adults aged 35–44 y reported that almost all the participants had a substantial number of dental and periodontal problems [20]. The same study also reported that 6.6% of the sample had missing teeth According to the WHO’s worldwide map of oral health, Iranian adults aged 35–44 y have a moderate level of dental caries [21]. The current study revealed that 8% of the participants were edentulous (no natural teeth), 15.5% had a removable partial denture, and 10% had a full (lower or upper) denture. The prevalence of edentulism among adults aged 51–65 y was 20.7%. A similar prevalence of edentulism among males aged 71–92 y was reported in a U.K. study [22]. However, there were some methodological differences between our studies and mentioned study in the field of gathering information and measurements where in that study physical examination of participants and brief oral health assessment were included [22].

In systematic review, a Chinese population aged 65 y had an average of 20 teeth [23]. In a Swiss health survey, by using self-report questionnaire, the prevalence of edentulous individuals among those aged 15–24 y and 65–71 y was 0.3 and 26.8%, respectively [24]. In a Turkish study the information were gathered through face to face administered questionnaire and an oral examination reported a higher rate (48%) of edentulism [25]. Although the burden of oral disease and tooth loss seems to increase with age, a substantial number of Iranian middle-aged adults experience oral health problems [26]. In the present study, more than one-third of middle-aged adults (i.e., aged 51–65 y) reported poor self-rated oral and gum health. These findings are similar to those found in a previous study, which reported poor self-rated oral health among Brazilian adults aged 20–59 y [26].

About one-third of the participants in the present study did not brush their teeth daily, whereas three-quarters of those aged 18–35 y brushed their teeth once a day or more. In a previous study on Iranian individuals aged 60–70 years, the authors reported that 20% of the participants brushed their teeth twice or more a day and that about half the participants brushed their teeth only once a day [27]. Our study also demonstrated that older participants were less likely to brush their teeth twice or more a day. Lack of knowledge on the importance of oral hygiene and cultural-specific factors may influence oral health behaviors of older adults [28]. As reported previously, younger adults seemed to be more familiar than older adults were with common and effective methods of preventive oral health care, possibly because of more interactions with community services, allowing them to obtain information about oral health preventive behavior [29].

In our study, only 23% of the participants reported using dental floss as part of routine oral hygiene. Recently, a systematic review and meta-analysis suggested that optimal oral health care for caries prevention and periodontal disease control required tooth brushing and dental flossing twice daily with fluoride toothpaste [30]. The findings of the present study highlight the need for education on the importance of dental flossing, which is just as important as tooth brushing, as it removes dental plaque from embrasures (interdental spaces) and proximal tooth surfaces.

In this study, about 30% of the participants had visited a dentist once in the previous 6 mo, and the most common reason for the visit was oral/dental pain or discomfort, gums, or mouth. Similarly, Burgette et al. reported that 57% of their American study participants had visited the dentist within the past year [31]. According to the results, the participants visited the dentist due to dental- or oral-related problems [31].. Thus, their dental health-seeking behavior was problem driven rather than preventive driven. Only 12.5% of the participants reported a routine check-up as the reason for their last visit to the dentist.

Mastication difficulty was the most common problem experienced by the participants, especially the older adults (51–65 y). It is assumed that tooth loss and the presence of dentures decreases chewing ability, dietary intake, and overall health. There is evidence that a mastication problem has a significant and negative impact on oral health-related QoL [32]. Among 44% of those aged 18–35 y, toothache was the most common problem associated with reduced QoL. In a study conducted in several European countries, 10% of the sample, which included denture wearers, reported reduced QoL because of oral and dental problems [33]. In another study, as the number of lost or decayed teeth increased, it was more difficult for participants to eat fruit (e.g., apples) [34].

In the present study, one-third of the participants consumed sweets with their meals every day and consumed tea with sugar daily. There is strong evidence for a significant association between a sugar-laden unhealthy diet and poor oral health [35, 36]. Poor oral health may have an indirect effect on general health by disrupting dietary intake. Oral health problems include edentulism, removable partial dentures that fit poorly, pain or cavities in teeth, and oral disease, all of which may compromise dietary intake by changing food choices [37–39]. These oral health problems are particularly important among older people, as evidenced by high levels of tooth loss, dental caries, and periodontal disease [40].

Most studies on oral hygiene have focused on specific populations, such as children [14] or pregnant women [41, 42], or have been conducted in oral health care centers and used convenience samples [43]. The present study adds to the literature by focusing on a large representative sample of the Iranian population. Nevertheless, the study has some limitations. First, it can be assumed that there is some selection bias, as not all eligible participants may have been at home during the interview visits. Second, only citizen of Tabriz were included in this study. Although the population of Tabriz is similar to that of other Iranian big cities where HCCs are present, it might be interesting for future studies to also focus on rural areas of Iran. Third, this study relied on self-reported oral health. Self-reports represent only the impressions of participants and are subject to response distortions (e.g., extreme or central tendency responses or socially desirable responses) [44]. A more appropriate way to measure the dependent and independent variables would have been through observations or reports by independent evaluators. It might be interesting for future studies to combine self-reports with official dental records. Furthermore, including participants from different social classes would enhance the applicability of the findings to the general population.

## Conclusions

This household survey showed that 8.1% of the participants were edentulous and 61% had experienced pain in the last 12 mo, 63% had good oral health, 65% of participants brushed their teeth daily, and 30% had visited a dentist in the previous 6 mo. Mastication difficulty was the main oral health problem among those aged 51–65 y, and about one-third of the sample consumed sweets and cakes on a daily basis. In the sample, only 23% performed dental flossing on a regular basis. Although the majority of Iranian adults considered their oral health status good, only a small number of participants visited their dentist regularly The oral health status of the participants indicates that although the majority of Iranian adults assess their teeth positively, only a small number of participants made routine dental visits. The findings can aid planning strategies for oral health care among the adult population.

## Data Availability

The data collection tools and datasets generated and/or analyzed during the current study are available from the corresponding author on reasonable request.

## Abbreviations

WHO: World Health Organization
OHQ: Oral Health Questionnaire
HCCs: Health care centers
QoL: Quality of life
